# Single-cell RNA-sequencing reveals a distinct population of proglucagon-expressing cells specific to the mouse upper small intestine

**DOI:** 10.1016/j.molmet.2017.07.014

**Published:** 2017-08-01

**Authors:** Leslie L. Glass, Fernando J. Calero-Nieto, Wajid Jawaid, Pierre Larraufie, Richard G. Kay, Berthold Göttgens, Frank Reimann, Fiona M. Gribble

**Affiliations:** 1 Metabolic Research Laboratories, Wellcome Trust MRC Institute of Metabolic Science, Addenbrooke's Hospital, Hills Road, Cambridge, CB2 0QQ, UK; 2 Wellcome Trust and MRC Cambridge Stem Cell Institute & Cambridge Institute for Medical Research, Addenbrooke's Hospital, Hills Road, Cambridge, CB2 0XY, UK

**Keywords:** Single cell RNA seq, GLP-1, PPG-cells, L-cells, Mass spectrometry, 5-HT

## Abstract

**Objectives:**

To identify sub-populations of intestinal preproglucagon-expressing (PPG) cells producing Glucagon-like Peptide-1, and their associated expression profiles of sensory receptors, thereby enabling the discovery of therapeutic strategies that target these cell populations for the treatment of diabetes and obesity.

**Methods:**

We performed single cell RNA sequencing of PPG-cells purified by flow cytometry from the upper small intestine of 3 GLU-Venus mice. Cells from 2 mice were sequenced at low depth, and from the third mouse at high depth. High quality sequencing data from 234 PPG-cells were used to identify clusters by tSNE analysis. qPCR was performed to compare the longitudinal and crypt/villus locations of cluster-specific genes. Immunofluorescence and mass spectrometry were used to confirm protein expression.

**Results:**

PPG-cells formed 3 major clusters: a group with typical characteristics of classical L-cells, including high expression of *Gcg* and *Pyy* (comprising 51% of all PPG-cells); a cell type overlapping with *Gip*-expressing K-cells (14%); and a unique cluster expressing *Tph1* and *Pzp* that was predominantly located in proximal small intestine villi and co-produced 5-HT (35%). Expression of G-protein coupled receptors differed between clusters, suggesting the cell types are differentially regulated and would be differentially targetable.

**Conclusions:**

Our findings support the emerging concept that many enteroendocrine cell populations are highly overlapping, with individual cells producing a range of peptides previously assigned to distinct cell types. Different receptor expression profiles across the clusters highlight potential drug targets to increase gut hormone secretion for the treatment of diabetes and obesity.

## Highlights

• Single cell RNAseq divides upper small intestinal PPG-cells into 3 major clusters.
• PPG-cell clusters exhibit distinct expression profiles for hormones and receptors.
• PPG-cells produce a variety of hormones detectable by mass spectrometry.
• A new mouse PPG-cell cluster expresses the markers *Tph1* and *Pzp*.

## 1 Introduction

Enteroendocrine preproglucagon-expressing PPG-cells (traditionally known as L-cells) secrete the gut hormones glucagon-like peptide 1 (GLP-1) and peptideYY (PYY) and are important regulators of glucose metabolism and appetite [1]. GLP-1 promotes glucose-dependent insulin secretion and satiety, and has been translated into therapies for type 2 diabetes and obesity through the development of long acting GLP-1 mimetics and inhibitors of dipeptidyl peptidase 4 (DPP4) [2]. PYY promotes satiety and is under investigation as a potential basis for new anti-obesity therapeutics [2].

These observations raised the prospect of developing treatments that promote the secretion of GLP-1 and other gut hormones. The strategy depends on understanding the different enteroendocrine cell populations and their relative expression of target receptors. Classically, a subpopulation of enteroendocrine cells defined by immunohistochemical staining for proglucagon-derived peptides (including GLP-1), often together with PYY, was designated as L-cells, referred to here as PPG-cells because of their characteristic expression of preproglucagon [3]. Recent studies investigating genetically tagged PPG-cells have shown that, as a population, they also express gut hormones previously thought to be expressed in distinct enteroendocrine cell types, including *Cck* (cholecystokinin, I-cells), *Sct* (secretin, S-cells), and *Gip* (glucose-dependent insulinotropic polypeptide, K-cells) [4]. However, it remained unclear whether cells expressing different hormone combinations represent fundamentally distinct cell populations.

Variability within the PPG-cell population is physiologically interesting because PPG-cell peptides show different post-prandial plasma profiles [5]. It has been proposed recently that within a single enteroendocrine cell, vesicle pools containing different hormones might be differentially responsive to stimuli [6], but it is also likely that expression of hormones, ion channels, transporters, and receptors varies between PPG-cell sub-populations. The aim of the present study was to use single cell RNA sequencing to determine whether PPG-cells can be sub-divided into clusters with distinct expression of gut hormones, receptors, and other nutrient sensing proteins.

## 2 Experimental procedures

### 2.1 Animal welfare and ethical statements

This research has been regulated under the Animals (Scientific Procedures) Act 1986 Amendment Regulations 2012 following ethical review by the University of Cambridge Animal Welfare and Ethical Review Body (AWERB). Mice were housed in individually ventilated cages with ad libitum access to water and chow. Mice were killed by cervical dislocation prior to tissue harvesting. Both male and female GLU-Venus mice [7] on a C57BL6 background were used.

### 2.2 Small intestine for FACS sorting

For single cell RNAseq, tissue was prepared from 3 male mice, aged 20–21 weeks. For FACS sorting, tissue pieces from the proximal 10 cm of the small intestine were stripped of the outer muscle layers. Tissue was chopped into 1–2 mm pieces and digested to single cells with 1 mg/ml collagenase in calcium-free Hanks Buffered Salt Solution (HBSS). Single cell suspensions were separated by FACS using an Influx Cell Sorter (BD Bioscience, USA). Side scatter, forward scatter, pulse width gates, and DAPI-staining were used to exclude debris and aggregates. Single fluorescent and non-fluorescent (control) cells were collected into individual wells of a 96-well plate containing lysis buffer 0.2% (v/v) Triton X-100 and 2 U/μl RNase inhibitor (Ambion) and stored at −80 °C.

### 2.3 Single-cell RNA sequencing (further details in supplementary material)

scRNA-seq analysis was performed using the Smart-seq2 protocol [8] as previously described [9]. Two mice were sequenced at low depth and one mouse at high depth. Cells with >20% reads mapping to mitochondrial genes were removed from downstream analyses. For the deeper sequenced sample, all cells with <750,000 reads mapping to endogenous RNA were excluded. Out of the 288 cells sorted across the 3 experiments, 94 and 95 passed quality control from the first 2 mice, and 75 cells passed from the deeper sequenced experiment with increased quality control stringency (78%). Data were normalized for sequencing depth and RNA quantity using size factors calculated on endogenous genes [10].

Clustering was performed on the dimensionality reduced tSNE co-ordinates using the R package, Mclust (v 5.1) using cells that passed QC from all 3 mice. This defined 6 populations of cells. Only clusters that contained cells from all 3 mice and only containing Venus positive cells were used for further analysis.

Differential expression analysis was limited to cells from the sample sequenced at higher depth. Differentially expressed genes were identified by performing pair-wise and unique comparisons between the 3 clusters using DESeq2 (v. 3.4). Hierarchical clustering was performed using the union of the top 15.

### 2.4 Cell collection for qPCR analysis

PPG-cells were isolated as above, with the variation that tissue pieces were incubated in 10 mM EDTA in Ca^2+^ free PBS for 5 min, then transferred to 10 ml Ca^2+^ free PBS and gently inverted to dissociate the villi. This was repeated 4 more times, with incubations 3–5 shaken more vigorously in PBS. The fractions were spun at 300 rcf, resuspended in HBSS, then re-centrifuged. For collecting mixed PPG-cell populations, these fractions were combined and digested in 1 mg/ml Collagenase in HBSS. For separate villus/crypt sorts, fractions 1–2 were retained separately to generate the villus-enriched fraction, and fractions 3–5 were filtered through 50 μm filters prior to centrifugation to generate crypt-enriched fractions. The two fractions were then separately incubated in collagenase. Digested tissue was centrifuged at 300 rcf, resuspended in HBSS with 0.1% BSA, spun again, and resuspended in ∼1 ml of HBSS supplemented with 10% FBS. Cell suspensions were FACS sorted using a MoFlo Beckman Coulter Cytomation sorter (FL, USA) to obtain populations of Venus-positive or Venus-negative (control) cells that were collected directly into lysis buffer for mRNA extraction. The successful separation of crypts from villi was confirmed by qPCR for *Apoa4* (villi) and *Defa5* (crypts) using cDNA prepared from Venus negative cells.

### 2.5 RNA extraction and quantitative RT-PCR

RNA extraction from FACS-sorted cells was performed using an RNeasy Micro Plus Kit (Qiagen). Samples were reverse transcribed according to standard protocols (Superscript III, Life Technologies). Quantitative RT-PCR was performed with 7900 HT Fast Real-Time PCR system (Applied Biosystems, Life Technologies), using TaqMan primer/probe sets supplied by Applied Biosystems. Expression was compared with that of β-actin measured on the same sample in parallel on the same plate, giving a CT difference (ΔCT) for β-actin minus the test gene. Mean, standard error, and statistics were performed on the ΔCT data and only converted to relative expression levels (2^ΔCT^) for presentation in the figures. All experiments were performed on 4 independently isolated cDNA samples (n = 4 mice). A table of TaqMan primer-probes used is listed in the supplementary material.

### 2.6 Immunofluorescence microscopy

Duodenum from GLU-Venus mice was fixed in 4% paraformaldehyde, dehydrated in 15% and 30% sucrose, and frozen in O.C.T. media (VWR, UK). Cryostat-cut sections (7–10 μm) were mounted directly onto polylysine-covered glass slides (VWR, Belgium). Slides were incubated for 1 h in blocking solution containing 5% donkey serum/1% BSA/0.05% Tween-20 and overnight in blocking solution with primary antisera of interest. A table of antisera used is listed in the supplementary material. Sections were washed with blocking solution and incubated with appropriate secondary antisera (AlexaFluor, Life Technologies, UK) diluted 1:300. Control sections were stained with secondary antisera alone. Sections were washed with PBS and mounted with Prolong Gold (Life Technologies, UK) before confocal microscopy (Leica TCS SP8 X, Germany).

### 2.7 Peptidomics

16,000–20,000 PPG-cells from the proximal 10 cm of the small intestine were FACS-sorted directly into a lysis solution of acetonitrile (ACN) 80% (v/v) in a Protein LoBind tube (Eppendorf). Proteins were precipitated and after centrifugation (5 min, 10,000 g), supernatants containing small proteins and peptides were collected and dried in a centrifugal evaporator (Eppendorf). Peptides were reduced and alkylated to break and cap disulphide bonds. Samples were analyzed using a ThermoFisher Ultimate 3000 nano LC system coupled to a Q-Exactive Plus Orbitrap mass spectrometer (Thermo Scientific, San Jose, USA). Peptides were separated on a nano easy column (Thermo Fisher Scientific) by a ramp of increasing concentration of ACN from 2 to 40% (v/v) in 0.1% formic acid solution over 145 min. A full scan range of 400–1600 *m*/*z* was performed, and the top 10 ions of each spectrum were selected for MS/MS analysis. The acquired data were analyzed using Peaks 8.0 software (Waterloo, ON, Canada) against the mouse Swissprot database (downloaded on 06/05/2016) with a no-digest setting, allowing the matching of endogenous peptides of up to 65 amino acids.

### 2.8 Data analysis

Statistical analysis was performed using GraphPad Prism 7 package (San Diego, CA, USA). Values were regarded as significant when p < 0.05.

## 3 Results

Venus-positive PPG-cells from the top 10 cm of the small intestines of two GLU-Venus mice [7] were collected by flow cytometry together with negative (non-fluorescent) control cells. Venus-positive cells were collected across a range of fluorescence intensities, including cells that were only dimly fluorescent (Figure S1). The transcriptional profiles of a total of 184 cells (159 Venus positive and 25 Venus negative) were obtained as previously described [8]. 3381 genes exhibited expression variability exceeding technical noise [11]. Dimensionality reduction was performed using the t-Distributed Stochastic Neighbour Embedding (t-SNE) method using only these 3381 genes. Different clusters could be observed that included cells from both mice. As expected, Venus positive and negative cells appeared in separate clusters. Venus-positive cells appeared as a heterogeneous population that could be separated into distinct subpopulations.

**Figure 1:**
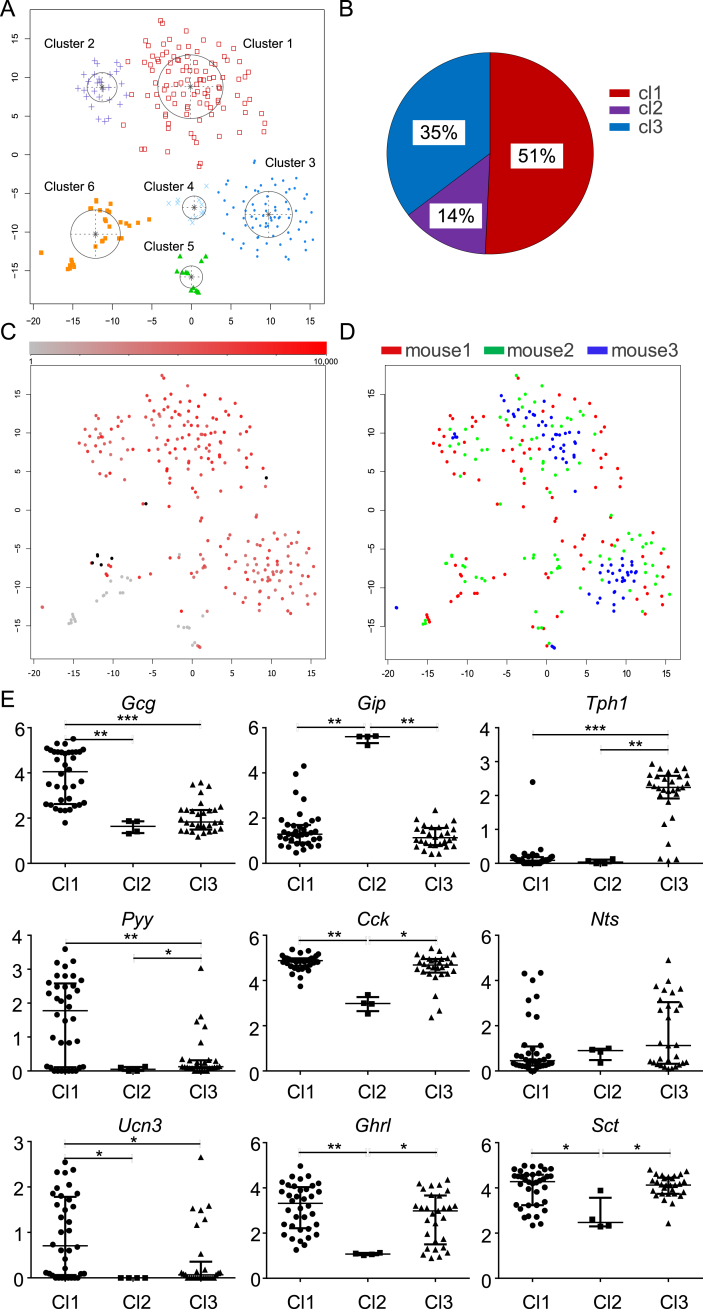
**PPG-cell clusters reflect hormone expression profiles**. A, C, D. tSNE analysis showing: Cluster allocation of the 259 included cells from all 3 mice. B. Relative proportions of cells located in Cl1-3, 203 cells in total. C. Venus fluorescence intensity of individual cells (redness intensity denotes relative brightness, grey = negative, black = no data), D. Donor origin of cells used in the single-cell RNA-Seq analysis (deeper sequenced mouse is depicted in blue). E. Relative gene expression levels of *Gcg*, *Gip*, *Tph1*, *Pyy*, *Cck*, *Nts*, *Ucn3*, *Ghrl*, and *Sct* in Cl1-3. All values displayed are the log_10_ (normalized counts), bars indicate median values and interquartile range. Statistical analysis was performed using Kruskal–Wallis with Dunn's multiple comparisons test. *p < 0.05, **p < 0.01, ***p < 0.001 between the indicated clusters.

Ninety six Venus positive cells from a third mouse were analyzed at greater depth, of which 75 passed strict quality control criteria (see methods). Combining all 259 cells from the 3 mice defined 976 highly variable genes that were used for tSNE analysis. This analysis generated 6 cell clusters (Cl1-Cl6) (Figure 1A–D), of which Cl5 and Cl6 contained Venus negative cells. Cl1-4 contained Venus-positive cells, of which Cl1-3 contained cells from all 3 mice. Cl4 contained <5% of cells, including one Venus-negative cell, but did not include any cells from the mouse sequenced at greater depth so was excluded from further analysis. Transcriptomic characteristics of Cl4 are shown in Figure S2. A few cells in Cl5 and Cl6 were Venus-positive and are likely to represent small groups of enterocytes containing at least one PPG-cell, as these could not be excluded completely during cell preparation and sorting.

Visual inspection of hormone expression profiles across the clusters revealed differential distributions of *Gcg*, *Gip*, *Pyy*, *Cck*, and *Tph1* (tryptophan hydroxylase-1 marking 5-HT producing cells), suggesting that the tSNE analysis separated cells that differ in their expression of hormonal genes. Subsequent quantitative comparisons between the clusters were performed using only data from mouse 3, in which the RNAseq read-depth was high (Figure 1E). Cl1 was distinguished by higher *Gcg* and *Pyy* expression than Cl2 and 3, and also expressed *Ucn3*. Cl2 had higher *Gip* expression than Cl1 and 3, but lower *Cck*, *Ghrl*, and *Sct*. Cl3 showed high expression of *Tph1*. Overall these data suggest that Cl1 contains archetypical “L-cells” expressing high levels of *Gcg* and *Pyy*, but still considerable detectable levels of *Cck*, *Sct*, and *Ghrl mRNA*; Cl2 contains *Gcg/Gip* dual positive cells; and Cl3 comprises a PPG-cell population expressing *Tph1*, but with lower levels of *Gcg* and *Pyy*.

**Figure 2:**
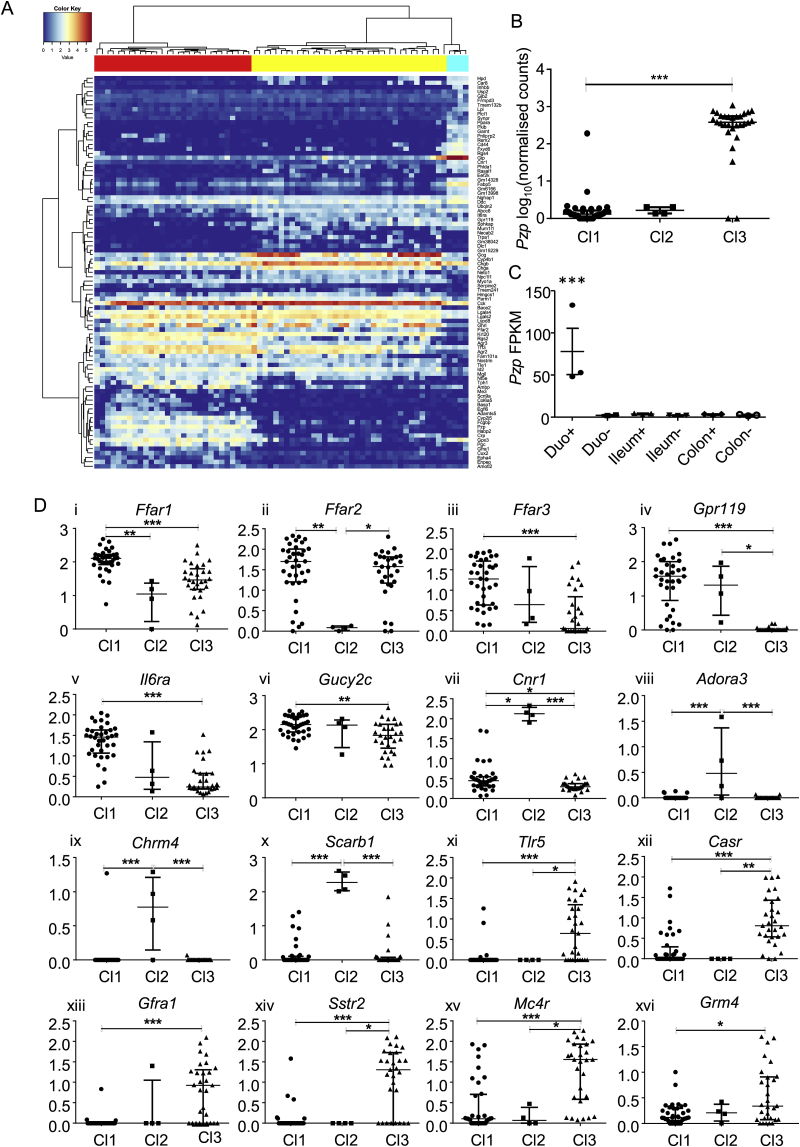
**Marker genes expressed across Cl1-3**. A. Hierarchical clustering of deeper sequenced cells using the union of the top 15 differentially expressed genes from the comparisons across CL1-3. Top coloring indicates cluster allocation: Cl1 in yellow, Cl2 in cyan and Cl3 in red. B. Log_10_ (normalized counts) for *Pzp*. Statistical analysis was performed using Kruskal–Wallis with Dunn's multiple comparisons test. C. Gene expression of *Pzp* measured by RNAseq analysis of cell populations isolated from PPG-cells and non PPG-cells from the duodenum (duo), ileum, and colon of Glu-Venus mice, expressed in fragments per kilobase per million reads. Proximal small intestinal PPG-cells were significantly different from all the other samples (p < 0.001) by pairwise comparison using Deseq2 (v3.5). D. Relative expression levels of *Ffar1, Ffar2, Ffar3, GPR119, IL6ra, Gucy2c, Cnr1, Adora3, Chrm4, Scarb1, Tlr5, Casr, Gfra1, Sstr2, Mc4r, Grm4* in single PPG-cells from the deep sequenced mouse. D i-vi represent genes characterizing Cl1, D vii-x characterize Cl2, and D xi-xvi characterize Cl3. Values displayed are the log_10_ (Normalized Counts). Bars indicate median values and interquartile range. Statistical analysis was performed using Kruskal–Wallis with Dunn's multiple comparisons test. *p < 0.05, **p < 0.01, ***p < 0.001 between the indicated clusters.

Genes differentially expressed in Cl1-3 were analyzed by hierarchical clustering and depicted in a heatmap (Figure 2A). Notably enriched in Cl1 were *Chga*, *Chgb*, *Gpr119*, and *Abcc8*. Cl2 selectively expressed genes previously identified in K-cells, including *Fabp5* and the cannabinoid receptor *Cnr1* [12], [13]. Pregnancy zone protein (*Pzp*) was a major marker of Cl3 (Figure 2B). RNAseq of PPG-cell populations isolated from different regions of the gut revealed that *Pzp* expression was found in PPG-cells from the proximal but not the distal small intestine, suggesting that Cl3 is specific to the upper small intestine (Figure 2C).

Further examination of genes that were differentially expressed between the clusters revealed a number of receptors previously implicated in enteroendocrine detection of nutrients and hormonal signals [1], as well as some receptors not previously characterized in gut endocrine cells (Figure 2D). Of note, Cl1 was enriched for *Ffar1*, *Ffar3*, *Il6ra*, and *Gpr119*. Cl2 was enriched for *Cnr1*, *Adora3*, and *Scarb1*. *Casr* and *Mc4r* were particularly prevalent in Cl3, as is also evident in data from all 3 mice (Figure S3). Between cells within a cluster, we observed variability in the expression of individual genes; however, cells exhibiting high levels of the Cl1 marker *Gpr119* tended to have lower levels of the Cl3 marker *Pzp* and vice versa (Figure S4).

**Figure 3:**
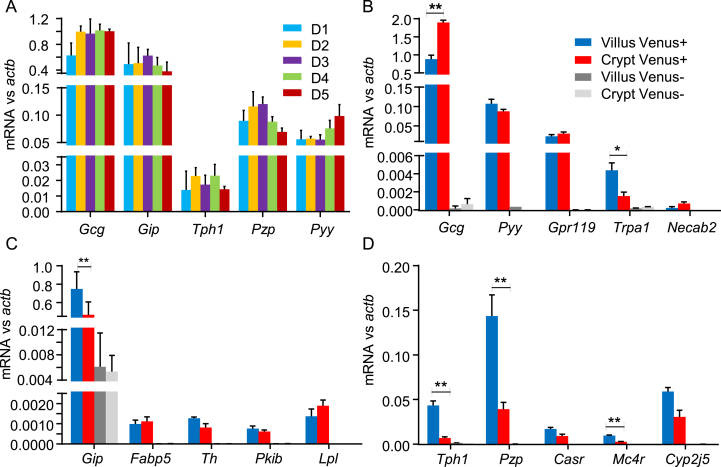
**Genes specific to Cl3 are enriched in villus PPG-cells**. A. qPCR analysis of *Gcg*, *Gip*, *Tph1*, *Pyy* and *Pzp* in PPG-cells purified from sequential 2 cm segments of the proximal small intestine (D1 to D5, running proximally to distally). No significant differences between segments were detected by ANOVA (n = 4). B-D. qPCR analysis of genes characteristic for Cl1 (B), Cl2 (C) and Cl3 (D) in Venus positive and negative cells collected from villus- and crypt-enriched fractions. Results are expressed as mean ± SEM converted to relative expression levels (2^ΔCT^) (n = 4). Villus vs crypt PPG-cells were compared by Student's paired t-test; *p < 0.05, **p < 0.01 for the comparisons indicated.

To examine whether Cl1-3 might represent PPG-cell populations characteristic of different positions along the 10 cm of small intestine examined, we measured expression of genes that distinguished the clusters by qPCR in Venus-positive cells purified by FACS from five sequential 2 cm segments of the upper small intestine. We found no evidence for statistically significant expression gradients of *Gcg*, *Gip*, *Tph1, Pzp*, *or Pyy* in PPG-cells along the longitudinal axis of the upper small intestine, although *Pzp* showed a trend of decreasing and *Pyy* a trend of increasing expression distally (Figure 3A).

We also examined whether any clusters were more prevalent in crypts or villi (Figure 3B–D), by sorting cells separately from crypt and villus enriched fractions and comparing expression of genes that distinguished the clusters by qPCR. *Gcg*, *Pyy*, *Gpr119*, *Trpa1*, and *Necab2* were used as markers for Cl1, but did not exhibit consistent patterns between PPG-cells from crypts and villi: *Gcg* was higher in crypt PPG-cells, *Trpa1* was higher in villi, and the other 3 markers did not differ between the two regions (Figure 3B). *Gip*, *Fabp5*, *Th*, *Pkib*, and *Lpl* were used to track Cl2, but similarly exhibited no consistent crypt/villus variation: *Gip* was more highly expressed in villus than crypt PPG-cells, but the other markers did not differ (Figure 3C). For Cl3, we examined *Tph1*, *Pzp*, *Casr*, *Mc4r*, and *Cyp2j5*. Three of these markers were significantly more highly expressed in PPG-cells from villi than crypts, and the other two showed a similar trend that was not significant (Figure 3D).

**Figure 4:**
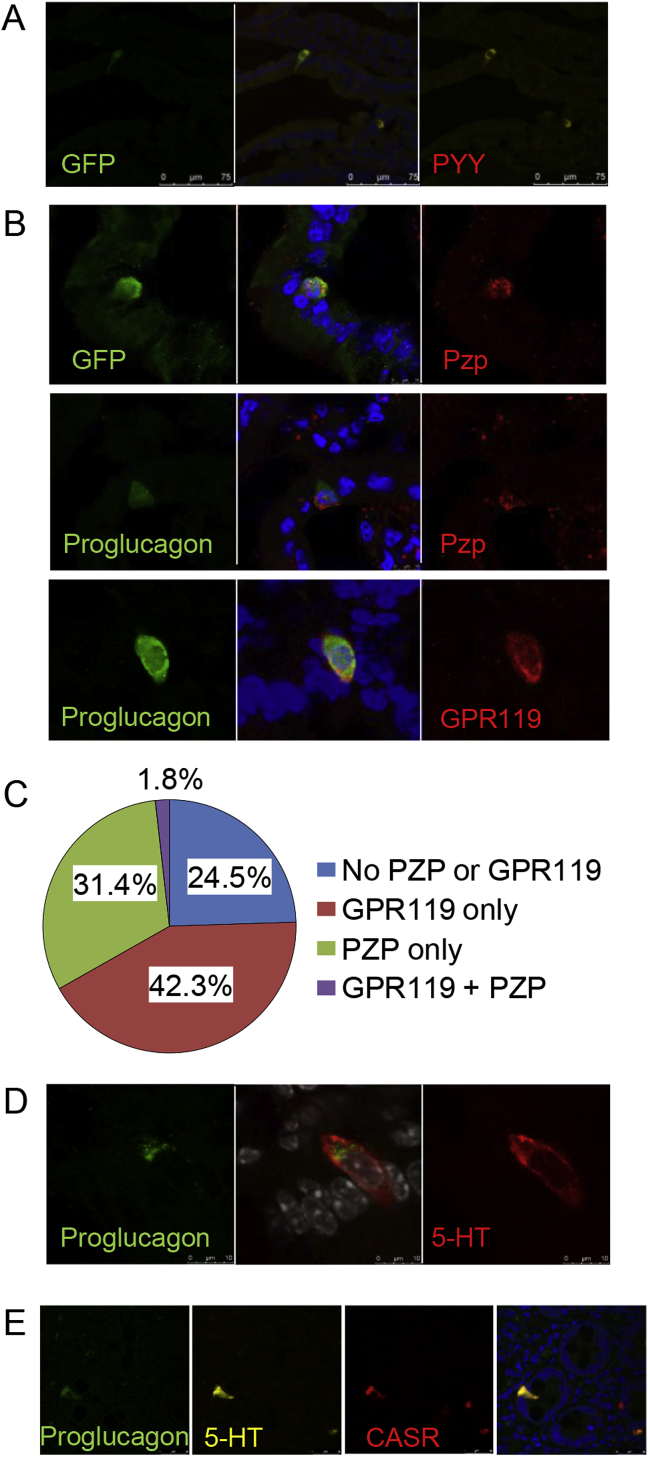
**Immunostaining of Cl1 and Cl3 markers**. A/B. GLU-Venus mouse duodenal sections were immunostained for GFP or proglucagon and PYY, PZP or GPR119 as indicated together with Hoechst stain (blue). C. Percentages of Venus positive cells co-staining for PZP and/or GPR119 as shown in B, calculated from a total of 220 cells counted. D. GLU-Venus mouse duodenal section stained for proglucagon (green) and 5-HT (red) and Hoechst (grey). E. Human duodenal sections co-stained for proglucagon (green), 5-HT (yellow), CASR (red) and Hoechst (blue).

To examine whether hormonal products predicted by gene expression in Figure 1E were detectable at the peptide level, we performed mass spec analysis of PPG-cells purified from the upper small intestine. Peptides from the prohormones of proglucagon, GIP, PYY, CCK, neurotensin, ghrelin, and secretin, but not Urocortin3 were detected (Figure S5). PYY production in PPG-cells from the top 2 cm of the small intestine was also confirmed by immunostaining (Figure 4A). As Cl1 was characterized by expression of *Gpr119*, and Cl3 by *Pzp*, we performed immunostaining of mouse duodenum to confirm the existence of the predicted Gcg/Venus positive cell populations producing PZP and GPR119 (Figure 4B and C) and to assess whether the two sub-populations could be differentiated at the level of protein expression. Co-staining indicated that very few Venus positive cells (<2%) co-expressed both GPR119 and PZP (Figure 4C), consistent with the cluster analysis. We confirmed production of 5-HT in mouse cells immunopositive for proglucagon (Figure 4D), and in human tissue slices, we demonstrated co-staining of the Cl3 marker CASR together with 5-HT and GLP-1 (Figure 4E).

## 4 Discussion

Enteroendocrine cells are traditionally classified according to the major one or two hormones they produce, but recent evidence has suggested that each enteroendocrine cell can express a number of different hormones, and that there is strong overlap between the cells producing GLP-1, PYY, CCK, GIP, secretin, and NTS [4], [14]. Single cell RNA sequencing has been used to study a variety of cell types, including pancreatic islet cells, neurons, and dissociated human intestinal organoids [15], [16], [17]. PPG-cells comprise less than 1% of the intestinal epithelium; thus, a previous study of intestinal organoids identified most of the cells as enterocytes and only a small number as enteroendocrine cells [17]. This analysis grouped enteroendocrine cells into a few small clusters, each including only a few cells, so did not produce an overall picture of PPG-cell diversity. Here we show that PPG-cells from the proximal small intestine can be divided into 3 major clusters with overlapping but distinct hormonal profiles.

Fourteen percent of Venus positive cells were located in a cluster characterized by high *Gip* expression (Cl2), consistent with our previous quantification of cells co-producing GLP-1 and GIP by FACS analysis [4]. The remainder of the Venus positive cells could be subdivided into two similar-sized groups, one with higher *Gcg* (Cl1) and the other with lower *Gcg* but high *Tph1* (Cl3). Only 4 cells from the deeply sequenced mouse were found in Cl2, so only the highly enriched genes in this group were identifiable with high probability. Among the characteristic Cl2 genes were several that we and others have previously identified as playing functional roles in K-cells, including *Cnr1* and *Fabp5* [12], [13]. Also appearing in this cluster were the scavenger receptor *Scarb1*, and lipoprotein lipase (*Lpl*), the roles of which for GLP-1 and GIP secretion remain unknown.

Cl1 exhibited significantly higher expression of *Gcg* and *Pyy* than the other two clusters, and also had the highest levels of *Chga* and *Chgb* (Figure S6), vesicular markers of enteroendocrine cells [4]. The high expression of *Gcg, Pyy*, and other gut hormones suggests that this cluster represents classical “L-cells”. We were surprised that the tSNE analysis did not separate this group into more clusters that might reflect subtle differences between cells co-expressing different hormonal combinations. It is possible, however, that analysis of PPG-cells from additional regions of the gut might identify more distinct clusters.

Cl1 was characterized by expression of a number of receptors and ion channels highlighted in previous studies, including *Gpr119*, *Ffar1*, *Il6ra*, *Gucy2c*, and *Trpa1*. *Gpr119* was detectable in ∼50% of PPG-cells, corresponding with our previous finding that about half of PPG-cells in primary culture exhibited cAMP responses to GPR119 agonists and that GLP-1 secretion was stimulated by GPR119 agonists [18]. *Ffar1*, by contrast, was found in almost all PPG-cells, although mean expression levels were highest in Cl1. This is a well-studied receptor for long chain free fatty acids, shown in a number of studies to contribute to fat-triggered GLP-1 secretion [19]. Cl1 was also enriched for *Il6ra*, which has been implicated in the modulation of PPG-cell function by interleukin-6 in response to high fat diet and exercise [20].

We were initially concerned that Cl3 might be an immature PPG-cell population, as it expressed lower levels of *Gcg* and chromogranins. Separate qPCR analysis of PPG-cells purified from crypts and villi, however, revealed that most genes that characterized Cl3 were more highly expressed in PPG-cells from villi than crypts. The predominant villus location of Cl3 markers suggests that this is a mature cell population, as immature cells should be located closer to their site of generation in the crypts. Alternatively, these cells might reflect a PPG-population gaining *Tph1* and *Pzp* expression with aging. The expression in this cluster of *Tph1* is consistent with a recent report that the majority of enterochromaffin cells in the mouse small intestine co-produce other gut hormones including PYY, GLP-1, and CCK [21]. The strongest marker of Cl3 was *Pzp*, an alpha2 macroglobulin of uncertain function [22]. Cl3 appeared distinct to the upper small intestine, as mRNA for *Pzp* was low in PPG-cells purified from the lower small intestine; a trend for higher expression in PPG-cells from the proximal compared to the distal intestine was also observed for another CL3 enriched gene *Tph1* (Figure S7), although this does not exclude that a relatively smaller sub-population of *Pzp and/or Tph1* co-expressing cells is present in the more distal PPG population.

Notable among the receptors found in Cl3 were the calcium sensing receptor (*Casr*) and melanocortin 4 receptor (*Mc4r*). We were surprised that *Casr* was more highly expressed in Cl3 than Cl1, as a number of studies have linked CASR activation to the secretion of GLP-1 [23], [24]. However, we demonstrated colocalization of CASR with 5-HT and GLP-1 in human intestinal slices as also reported previously [25], and exposure of human colonic tissue to a phenylalanine/tryptophan mixture has been reported to increase pCAMKII labeling in 5-HT and GLP-1 expressing cells [25]. *Mc4r* expression has been reported previously in colonic PPG-cells, where its activation was linked to PPG-cell secretion [26].

## 5 Conclusions

In summary, we conclude that upper small intestinal PPG-cells can be separated into at least 3 major clusters that exhibit differential expression of *Gcg*, *Cck*, *Pyy*, *Gip*, and *Tph1*. Receptor and ion channel expression profiles differed across the clusters suggesting that these PPG-cell sub-populations likely contribute to the differential responsiveness of gut hormones to nutritional and local signals.

## Author contributions

LLG and FJC-N performed experiments, analyzed data and helped to write the manuscript. PL and RGK performed population RNAseq and mass spec analyses. WJ analyzed data. BG took responsibility for single cell RNAseq protocols and bioinformatics analysis. FR and FMG jointly designed and supervised the project and wrote the manuscript. All authors approved the final manuscript.
