# Supplementary material for: Single-cell RNA-sequencing reveals a distinct population of proglucagon-expressing cells specific to the mouse upper small intestine
Source: Mol Metab. 2017 Aug 1;6(10):1296–303. doi: 10.1016/j.molmet.2017.07.014 (PMC5641633; doi:10.1016/j.molmet.2017.07.014)
Supplement: Supplementary file 1 [file mmc1.pdf]

Supplementary methods

Single-cell RNA sequencing – additional detail

scRNA-seq analysis was performed using the Smart-seq2 protocol,(1) as previously described (2). In brief, libraries were prepared using an Illumina Nextera XT DNA preparation kit and sequenced on an Illumina Hi-Seq 2500. Reads were mapped to the *Mus musculus* genome (EMSEMBL GRCm38.p4 Release 81) and ERCC sequences using GSNAP (version 2014-10-07). HTseq-count was used to count reads mapped to each gene (3). Cells with >20% reads mapping to mitochondrial genes were considered low quality and removed from downstream analyses. The deeper sequenced sample underwent more stringent quality control and, besides the above criteria, all cells with <750,000 reads mapping to endogenous RNA were excluded. Out of the 288 cells sorted across the 3 experiments, 94 and 95 passed quality control from the first 2 mice, and 75 cells passed from the deeper sequenced experiment with increased quality control stringency (78%). Data were normalised for sequencing depth and RNA quantity using size factors calculated on endogenous genes (4).

To identify highly variable genes we fitted the squared coefficient of variation as a function of the mean normalised ERCC counts (5). To minimise the skewing effect of low expressed genes, we used only ERCCs with a mean normalised count >80th centile. Genes with an adjusted p-value (Benjamini-Hochberg method) less than 0.1 were considered significant. This resulted in 976 highly variable genes.

tSNE dimensionality reduction was performed using the Rtsne package but with a pre-calculated dissimilarity matrix. Clustering was performed on the dimensionality reduced tSNE co-ordinates using Gaussian mixture models from the R package, Mclust (v 5.1). The best performing model was selected using the Bayesian Information Criterion. This defined 6 populations of cells. Only clusters that contained cells from all 3 mice and only containing Venus positive cells were used for further analysis.

To improve detection and reduce noise, differential expression analysis was limited to cells from the sample sequenced at greater depth. Differentially expressed genes were identified by performing pair-wise and unique comparisons between the 3 clusters using DESeq2 (v. 3.4) for all genes with a false discovery rate (Benjamini-Hochberg) of 0.05. Genes thus identified were used to manually assign cluster identity.

Hierarchical clustering was performed using the union of the top 15 genes from each of the comparisons across CL1-3. Clustering was performed using the ward.D2 method implemented in the heatmap.2 function (gplots v. 3.0.1) and branches were re-ordered using the order.optimal function (cba v. 0.2-17) all in R.

Table of qPCR primer sets

Gcg	Mm01269055_m1
Pyy	Mm00520716_g1
Necab2	Mm00475387_m1
Trpa1	Mm01227437_m1
Gpr119	Mm00731497_s1
Gip	Mm00433601
Th	Mm00447557_m1
Pkib	Mm00435846_m1
Tph1	Mm01202614
Pzp	Mm00431533_m1
Mc4r	Mm00457483_s1
Casr	Mm00443375_m1
cyp2j5	Mm00487292_m1
Defa5	Mm00651548_g1
Alpi	Mm01285814_g1
Apoa4	Mm00431814_m1
Actb	Mm02619580

Table of Antibodies used for immunofluorescence staining

Target	Species	Dilution	Source	Cat.#
GFP	Rabbit	1:1000	Abcam	ab290
PZP	Goat	1:200	Santa Cruz	SC-8516
GPR119	Rabbit	1:100	Abcam	ab75312
Casr	Mouse	1:500	Abcam	ab19347
Proglucagon	Goat	1:200	Santa Cruz	SC-7782
5-HT	Rabbit	1:10,000	Immunostar	20080
Proglucagon	Goat	1:100	Santa Cruz	SC-26637
Proglucagon	Rabbit	1:100	Santa Cruz	SC-13091
PYY	Rabbit	1:200	Abcam	ab22663

Figure S1

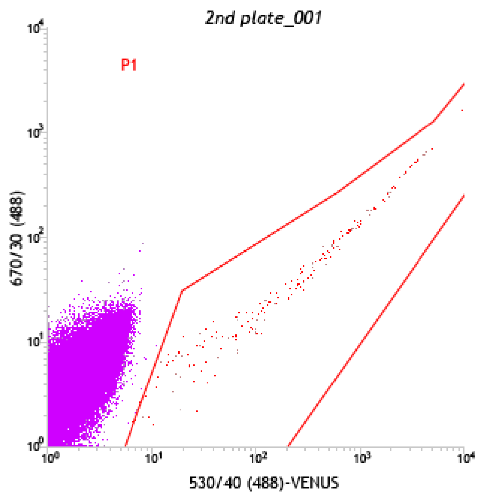

Figure S1. Gating used for purification of Venus positive cells. Additional gates included side scatter, forward scatter and pulse width. DAPI-staining was used to exclude debris and aggregates.

Figure S2

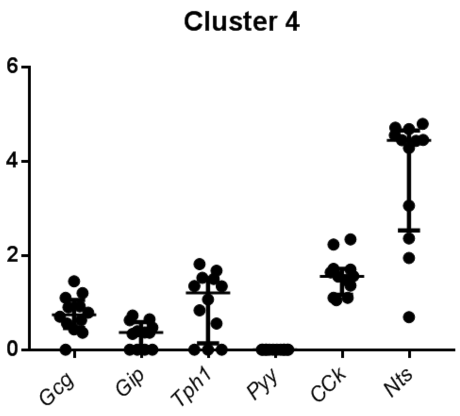

Figure S2 Log10(normalised counts) for Cluster 4 cells for classical enteroendocrine hormonal marker genes.

Figure S3

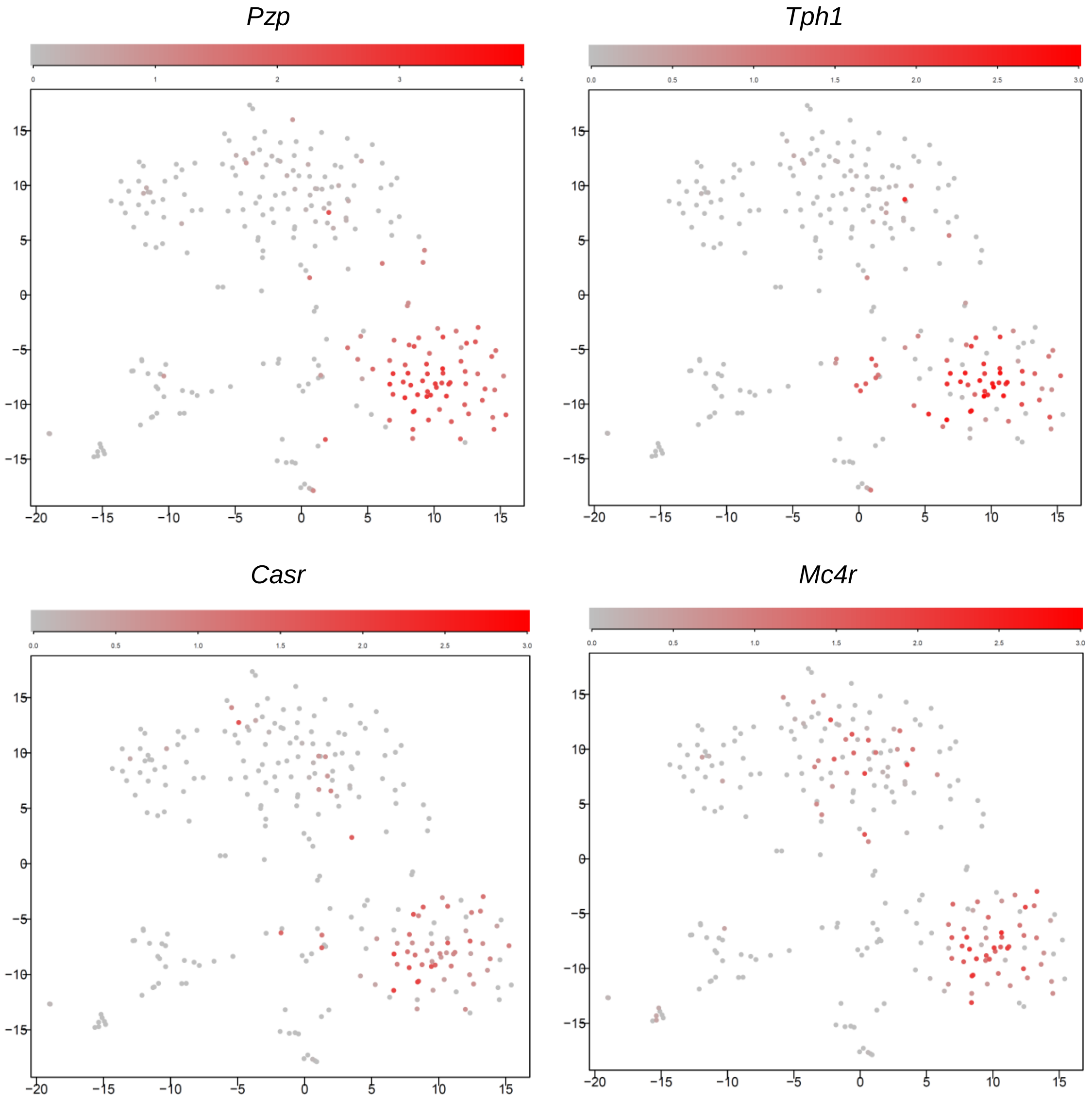

Figure S3. Cluster 3 gene expression: Semi-quantitative mRNA expression of individual cells for the genes indicated. Higher red intensity denotes higher mRNA expression, grey = negative

Figure S4

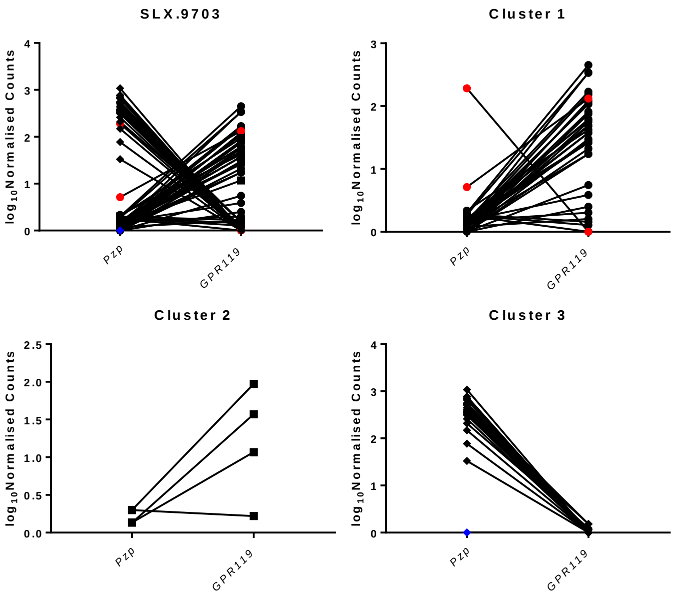

Figure S4. Expression of Gpr119 and Pzp in individual cells. Y-axes show $\log_{10}$(normalised counts). SLC9703 represents the mouse sequenced at high depth and the individual cells from the mouse are shown in separate sub-figures according to their cluster designation. CL1 outliers expressing uncharacteristically high levels of Pzp are highlighted in red and a CL3 outlier, neither expressing Pzp or GPR119 is highlighted in blue.

Figure S5

GCG (*Gcg*)

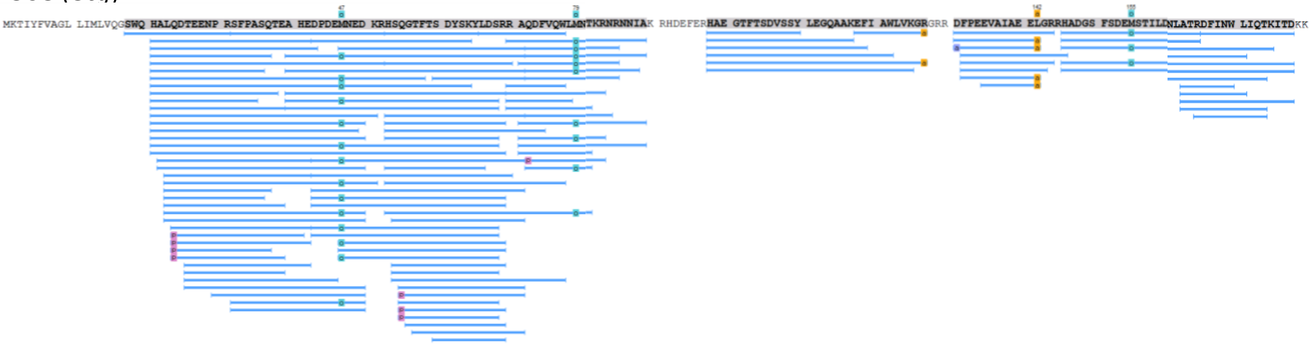

GIP (*Gip*)

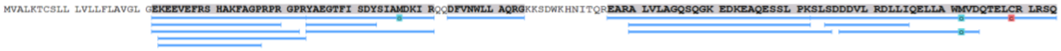

PYY (*Pyy*)

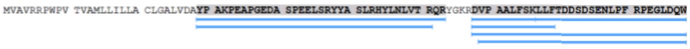

CCKN (*Cck*)

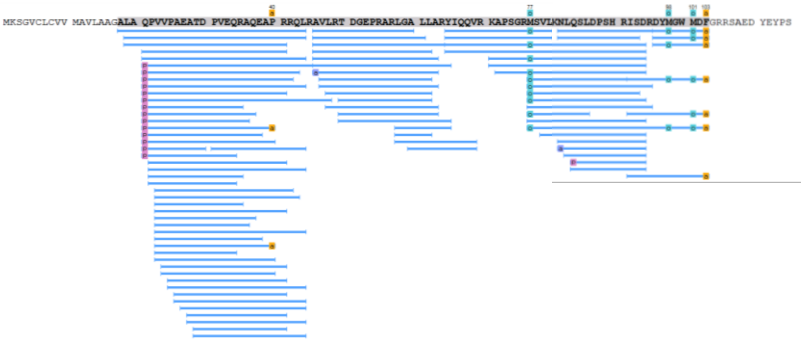

NEUT (*Nts*)

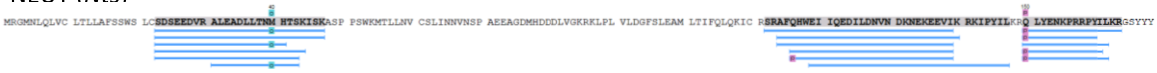

GHRL (*Ghrl*)

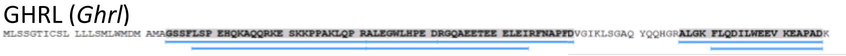

SECR (*Sct*)

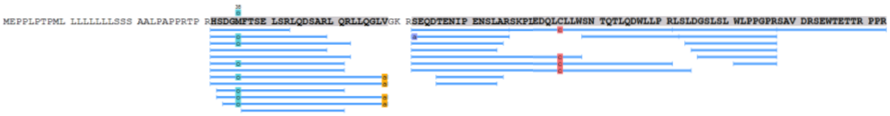

Figure S5: Peptidomics of GLU-Venus positive mice.. Representation of all the peptides identified by LC-MS/MS matching the canonical sequence of the prohormones expressed in the GLU-Venus positive cells (named by their UniprotKB / SwissProt identifier, with the associated gene indicated). Each bar shows a sequence that has been matched at least once with a high confidence (-10lgP > 27) by an ion analysed by MS/MS. Post-translational modifications searched for are color-coded: purple: N-terminal pyro-glutamate, blue: N-terminal acetylation, cyan: oxidation, yellow: C-terminal amidation and red: carbamidomethylation).

Figure S6

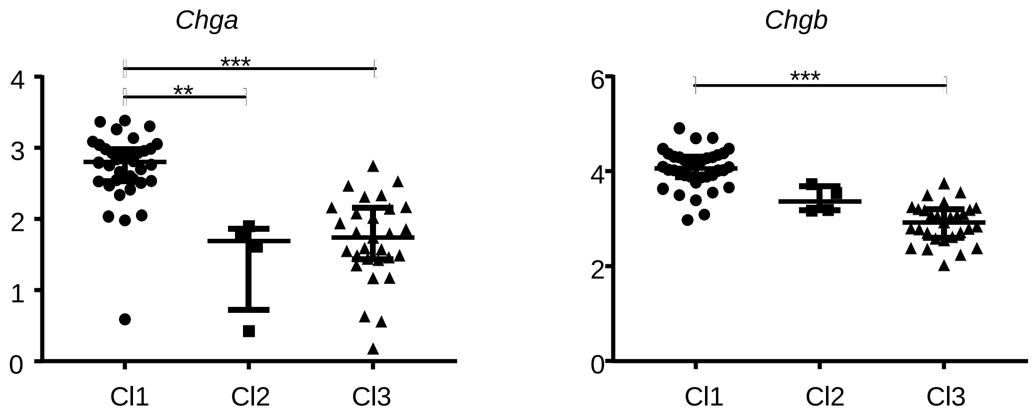

Figure S6 Expression of *Chga* and *Chgb* in CL1-3 by single cell RNAseq (expressed as $\log_{10}$(normalised counts))

Figure S7

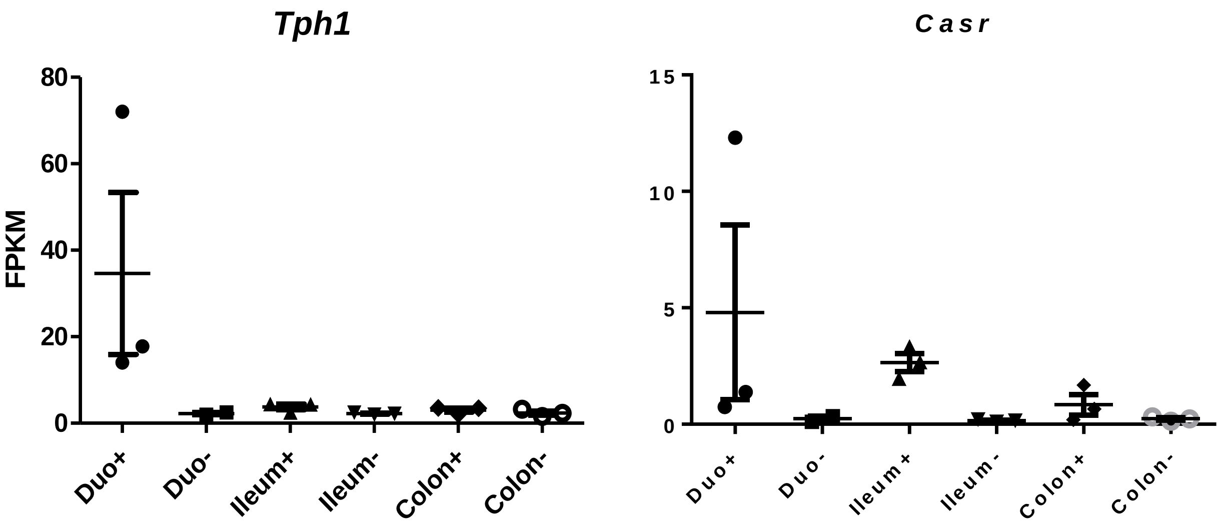

Figure S7: Gene expression of *Tph1* and *Casr* measured by RNAseq analysis of cell populations isolated from Venus+ and Venus- cells from the top 10 cm of the small intestine (duo), lower 10 cm of the small intestine (ileum) and colon of Glu-Venus mice, expressed in fragments per kilobase per million (FPKM)

Suppl material references

1. Picelli S, Faridani OR, Björklund AK, Winberg G, Sagasser S, Sandberg R: Full-length RNA-seq from single cells using Smart-seq2. Nat Protoc 2014;9:171-181
2. Scialdone A, Tanaka Y, Jawaid W, Moignard V, Wilson NK, Macaulay IC, Marioni JC, Göttgens B: Resolving early mesoderm diversification through single-cell expression profiling. Nature 2016;535:289-293
3. Anders S, Pyl PT, Huber W: HTSeq--a Python framework to work with high-throughput sequencing data. Bioinformatics 2015;31:166-169
4. Anders S, Huber W: Differential expression analysis for sequence count data. Genome Biol 2010;11:R106
5. Brennecke P, Anders S, Kim JK, Kołodziejczyk AA, Zhang X, Proserpio V, Baying B, Benes V, Teichmann SA, Marioni JC, Heisler MG: Accounting for technical noise in single-cell RNA-seq experiments. Nat Methods 2013;10:1093-1095
